# Cinematic Rendering in Anatomy Education: Revolutionizing Visual Learning

**DOI:** 10.7759/cureus.99250

**Published:** 2025-12-15

**Authors:** Pradosh Kumar Sarangi, Ravi Kant Narayan, Kondaveeti Nikhileswar, Swaha Panda

**Affiliations:** 1 Radiodiagnosis, All India Institute of Medical Sciences, Deoghar, Jharkhand, IND; 2 Anatomy, All India Institute of Medical Sciences, Bhubaneswar, Odisha, IND; 3 Otolaryngology, All India Institute of Medical Sciences, Deoghar, Jharkhand, IND

**Keywords:** anatomy education, cinematic rendering, immersive learning, medical visualization, radiology-based teaching

## Abstract

Cinematic Rendering (Siemens Healthliners, Erlangen, Germany) is an advanced imaging technique that converts radiological data into highly realistic 3D visualizations, offering a transformative tool for anatomy education. Traditional teaching methods often lack spatial clarity and interactivity, limiting students’ understanding of complex anatomical relationships. Cinematic Rendering overcomes these challenges by providing lifelike detail, dynamic exploration, and the ability to visualize real patient cases and pathological changes. This enhances knowledge retention, spatial comprehension, and clinical reasoning. The technology also supports remote collaboration and offers an ethical alternative to cadaver-based teaching. As medical schools begin adopting Cinematic Rendering, emerging virtual and augmented reality applications are expected to further expand its educational impact. Overall, Cinematic Rendering represents a significant advancement in anatomical teaching, delivering immersive, accurate, and clinically relevant learning experiences.

## Editorial

Understanding human anatomy is an essential foundation in medical education. To understand the complexity of the human body, medical students have traditionally depended on textbooks, cadaver dissections, plastic models, and conventional two-dimensional imaging. This teaching of human anatomy has remained fundamentally unchanged for more than a century. While these methods have proven educational value, multiple studies have consistently shown that students struggle with spatial perception, depth understanding, and the translation of anatomical knowledge into clinical practice when trained mainly with 2D resources [1]. However, as technology has advanced, a groundbreaking visual technique called Cinematic Rendering (Siemens Healthliners, Erlangen, Germany) has evolved. This innovative technology has revolutionized anatomy education by presenting students with a realistic and engaging learning experience. In this post, we will examine the notion of Cinematic Rendering and how it affects anatomy instruction [1,2].

What is Cinematic Rendering?

Cinematic Rendering is a cutting-edge imaging approach that combines radiological data with powerful visualisation algorithms to produce astonishingly lifelike and detailed three-dimensional renderings of anatomical structures. Unlike standard rendering approaches, Cinematic Rendering generates pictures that closely resemble high-quality photos or movie stills, achieving previously unimaginable levels of realism. Cinematic Rendering is derived from standard computed tomography (CT) and magnetic resonance imaging (MRI) datasets but applies complex lighting, shadowing, and ray-tracing algorithms to generate hyper-realistic three-dimensional (3D) visualizations [3,4].

It was developed by Siemens Healthineers and has received FDA approval for clinical use [3,5-7]. Although Cinematic Rendering follows the same fundamental steps as conventional volume rendering, its substantially more complex lighting and shading algorithms require higher computational power and result in longer post-processing times. The technique generates highly photorealistic 3D representations with superior depth, contour, and shape perception compared to traditional rendering methods. Cinematic Rendering has several potential applications, including anatomy teaching, patient education, surgical planning (particularly in cardiac, vascular, and trauma surgery), and enhanced disease detection. Additionally, Cinematic Rendering may serve as an effective alternative to 3D printing in settings where printed anatomical models are unavailable [3,6,7].

**Figure 1:**
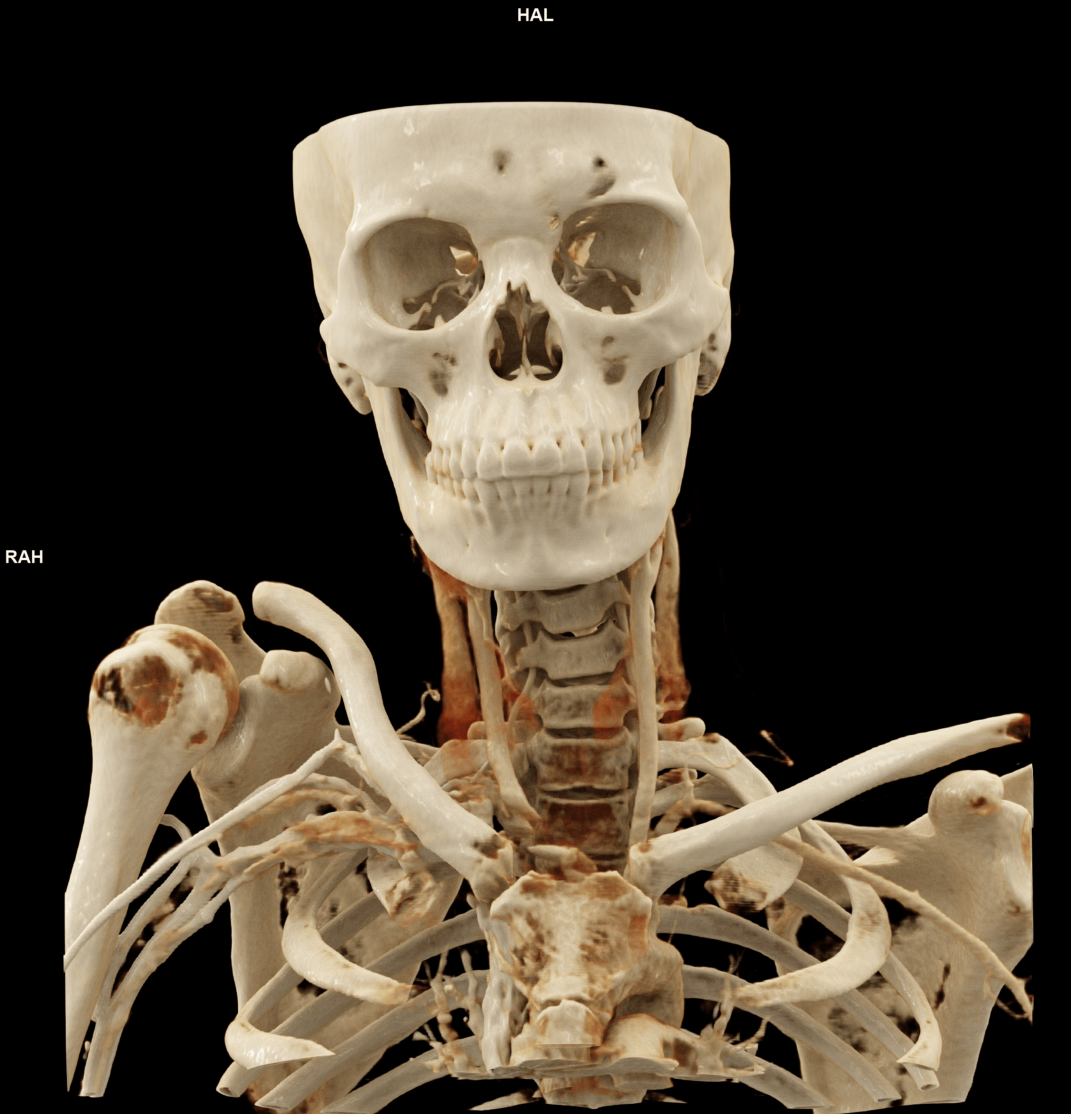
Cinematic Rendering of the face, neck, and upper chest generated using the syngo.via platform (Siemens). Image created in the Department of Radiodiagnosis, All India Institute of  Medcial Sciences, Deoghar.

A representative image of Cinematic Rendering from our institute is provided in Figure 1.

Current issues in anatomy education

Current anatomy education faces several important challenges, including restrictive teaching methods that rely heavily on traditional lecture formats and standardized models and illustrations, which often limit students’ ability to fully appreciate anatomical variability. In addition, the reliance on two-dimensional visualization makes it difficult to accurately represent the true spatial complexity of human anatomy, affecting depth perception and three-dimensional understanding. Furthermore, there is often insufficient inclusion of real patient cases in routine teaching, which reduces opportunities for students to connect anatomical knowledge with clinical application and real-world relevance.

Cadaveric dissection remains highly valued by students and faculty but faces reduced availability, ethical concerns, regulatory constraints, and high maintenance costs. In several regions, there is an outright shortage or difficulty in sourcing bodies, forcing greater reliance on prosections, models, and digital tools, with ongoing debate about the impact on clinical competence and professionalism.

It has been demonstrated that structured, image-based, and clinically oriented learning environments significantly improved students’ ability to identify anatomical structures, interpret cross-sectional images, and integrate anatomical knowledge with imaging modalities, while also enhancing teamwork and engagement [8]. Furthermore, large-scale survey data from 35 medical schools across six regions revealed marked variability in students’ anatomy study preferences, learning strategies, and resource utilization, influenced not only by academic year but also by regional educational contexts. This heterogeneity highlights the limitations of standardized teaching models and supports the need for adaptable, visually rich educational tools capable of accommodating different cognitive and spatial learning approaches [9].

Benefits of Cinematic Rendering in anatomy education

Realism and Accuracy

Cinematic Rendering allows pupils to see anatomical structures with unmatched realism. The realistic look of organs, tissues, and structures enables a better comprehension of their spatial interactions and complexities. This increased realism improves the learning process and aids in information retention [5].

Interactive Exploration

Students may use Cinematic Rendering to examine anatomical structures from various viewpoints, zoom in and out, and change the visualisations in real time. This interaction encourages active learning and assists students in developing a thorough grasp of the human body [1,2].

Clinical Context

Cinematic Rendering also strengthens the clinical relevance of anatomy education by allowing students to study structures in real clinical contexts. By simulating various diseases and pathological conditions, students can observe how anatomy is altered in disease states. This visual exposure sharpens their diagnostic reasoning and supports the development of critical thinking skills required for patient management [5].

Collaboration and Accessibility

Cinematic Rendering is a powerful tool for collaborative learning since it can be readily shared and accessible across several platforms. Remote collaboration allows students and educators to debate challenging issues and share their results, promoting a collaborative and engaging learning environment [3].

Ethical Considerations

Finally, Cinematic Rendering addresses important ethical concerns associated with the traditional use of cadavers in anatomy teaching. By generating highly detailed and accurate visualizations without the need for human specimens, it offers an ethical alternative that respects donor dignity while still ensuring high-quality anatomical education. This approach also helps overcome limitations related to the availability, cost, and maintenance of cadaveric material [4].

A randomized crossover study from Germany showed that Cinematic Rendering reduced anatomy interpretation time by 65.6% compared with conventional CT, was rated superior for musculoskeletal and vascular anatomy, and was widely accepted by students as a valuable tool for medical education [4]. A survey-based study of 120 first-year medical and dental students from Germany found that over 95% perceived Cinematic Rendering-featured anatomy lectures as helpful for understanding anatomy, with 85% reporting improved learning and strong acceptance for future self-study use [10].

Implementation and future directions

Cinematic Rendering is a new tool in anatomy instruction, but its potential is enormous. Medical schools and institutes increasingly use this unique technique to improve their courses. Integrating Cinematic Rendering into anatomy classes can dramatically improve student engagement, information retention, and critical thinking abilities.

Advances in virtual reality (VR) and augmented reality (AR) technology may further revolutionise anatomy instruction. Consider the possibility of students entering a virtual anatomy lab, interacting with virtual organs, and doing virtual dissections in a realistic and immersive setting. Such developments bode well for the future of anatomical teaching [3,5].

Despite its promise, Cinematic Rendering is not without limitations. High-end computing requirements, software licensing costs, and the need for faculty training remain barriers to widespread adoption [7]. Moreover, Cinematic Rendering should be viewed as a complementary tool rather than a complete replacement for cadaveric dissection, especially for tactile learning and surgical skill acquisition. A hybrid educational model that integrates cadaver-based teaching with Cinematic Rendering-based visualization may offer the most balanced and effective approach.

Conclusion

Cinematic Rendering has ushered in a new age in anatomy instruction, providing students with unparalleled realism and involvement. This technology has altered how medical students study and comprehend human anatomy. Cinematic Rendering improves knowledge retention, critical thinking, and clinical reasoning abilities by giving a more realistic and immersive experience. As this technology advances, it has the potential to significantly improve medical education, eventually benefiting healthcare workers and the people they serve.

## References

[1] Webb AL, Choi S (2014). Interactive radiological anatomy eLearning solution for first year medical students: development, integration, and impact on learning. Anat Sci Educ.

[2] Fellner FA, Engel K, Kremer C (2017). Virtual anatomy: the dissecting theatre of the future—implementation of cinematic rendering in a large 8 K high-resolution projection environment. J Biomed Sci Eng.

[3] Dappa E, Higashigaito K, Fornaro J, Leschka S, Wildermuth S, Alkadhi H (2016). Cinematic rendering - an alternative to volume rendering for 3D computed tomography imaging. Insights Imaging.

[4] Binder JS, Scholz M, Ellmann S, Uder M, Grützmann R, Weber GF, Krautz C (2021). Cinematic rendering in anatomy: a crossover study comparing a novel 3D reconstruction technique to conventional computed tomography. Anat Sci Educ.

[5] Rowe SP, Meyer AR, Gorin MA, Johnson PT, Fishman EK (2018). 3D CT of renal pathology: initial experience with cinematic rendering. Abdom Radiol (NY).

[6] (2026). Cinematic rendering in medical imaging. https://www.siemens-healthineers.com/en-in/digital-health-solutions/cinematic-rendering.

[7] Eid M, De Cecco CN, Nance JW Jr (2017). Cinematic rendering in CT: a novel, lifelike 3D visualization technique. AJR Am J Roentgenol.

[8] Ogut E, Yildirim FB, Senol Y, Senol AU (2025). Comprehensive evaluation of the educational impact and effectiveness of specialized study modules in cross-sectional anatomy: a study on student engagement and learning outcomes. BMC Med Educ.

[9] Barut C, Ogut E, Karaer E, Yavuz M (2025). Anatomy study preferences of medical students in relation to gender, academic year and geographical distribution: considerations for anatomy education approaches. Bratisl Med J.

[10] Binder J, Krautz C, Engel K, Grützmann R, Fellner FA, Burger PH, Scholz M (2019). Leveraging medical imaging for medical education - A cinematic rendering-featured lecture. Ann Anat.

